# A Meta-Analysis of the Impact of Using Angiotensin-Converting Enzyme Inhibitors (ACEIs) or Angiotensin II Receptor Blockers (ARBs) on Mortality, Severity, and Healthcare Resource Utilization in Patients with COVID-19

**DOI:** 10.3390/arm93010004

**Published:** 2025-02-18

**Authors:** Ruijuan Li, Jie Zhang, Liang Ren

**Affiliations:** Department of Forensic Medicine, Tongji Medical College of Huazhong University of Science and Technology, Wuhan 430032, China; m202375896@hust.edu.cn (R.L.);

**Keywords:** clinical medicine, coronavirus disease 2019, severe acute respiratory syndrome coronavirus 2, angiotensin-converting enzyme inhibitors, angiotensin II receptor blockers

## Abstract

**Highlights:**

This study addresses a critical question about the impact of ACEIs and ARBs on COVID-19, offering valuable insights through a robust meta-analysis. Its strength lies in professional statistical evaluation across diverse datasets, resolving prior inconsistencies.

**What are the main findings?**

- The use of ACEIs and ARBs presents both advantages and disadvantages for patients with COVID-19.
- The utilization of ACEIs and ARBs does not demonstrate a substantial correlation with mortality, severity, or healthcare resource utilization in patients with COVID-19.

**What is the implication of the main finding?**

- The utilization of ACEIs and ARBs in patients diagnosed with COVID-19 is associated with benefits that outweigh the potential drawbacks.
- The utilization of ACEIs and ARBs has been shown to be safe medical practice.

**Abstract:**

Objective: The primary objective of this study is to explore the potential link between the utilization of angiotensin-converting enzyme inhibitors (ACEIs) or angiotensin II receptor blockers (ARBs) and its impact on mortality, disease severity, and healthcare resource utilization in individuals diagnosed with COVID-19. We aim to establish a solid theoretical foundation for safe and effective clinical medications. Methods: We conducted a comprehensive search of various databases, including CNKI, PubMed, Science, Cell, Springer, Nature, Web of Science, and Embase. We also traced the literature of the included studies to ensure a thorough analysis of the available evidence. After applying a set of inclusion and exclusion criteria, we ultimately included a total of 41 articles in our analysis. To determine the overall effect size for dichotomous variables, we used the Mantel–Haenszel odds ratio in random effect models. For continuous variables, we calculated the inverse variance SMD using random effect models. To assess the outcomes and heterogeneity, we considered *p*-values (*p* < 0.05) and I^2^ values for all outcomes. We performed multivariate and univariate meta-regression analyses using the maximum likelihood approach with the CMA 3.0 software. Results: The results of our analysis indicated that the use of ACEIs or ARBs did not significantly influence mortality (OR = 1.10, 95% CI 0.83–1.46, *p* = 0.43, I^2^ = 84%), severity (OR = 0.99, 95% CI 0.68–1.45, *p* = 0.98, I^2^ = 84%), or healthcare resource utilization (SMD = 0.03, 95% CI 0.06–0.12, *p* = 0.54, I^2^ = 37%) in patients with COVID-19 compared to those not taking ACEIs or ARBs. The multivariate meta-regression analysis model explained 63%, 31%, and 100% of the sources of heterogeneity for the three outcome indicators. Conclusions: The use of ACEIs and ARBs is not significantly correlated with mortality, severity, or healthcare resource utilization in patients with COVID-19, indicating safe clinical use of the medications.

## 1. Introduction

Severe acute respiratory syndrome coronavirus-2 (SARS-CoV-2) is the pathogen of COVID-19 [1]. It was declared a pandemic on 30 January 2020 [2,3]. The COVID-19 pandemic has overwhelmed the healthcare systems of most countries and led to substantial economic losses. As of 5:34 p.m. Central European Time (CET) on 1 December 2022, COVID-19 cases, including 6,615,258 deaths, have been reported to the World Health Organization (WHO), and as of 29 November 2022, a total of 13,042,112,489 doses of vaccines have been vaccinated [4]. SARS-CoV-2 is usually transmitted by respiratory tract. The main symptoms include fever, cough, weakness, sputum, hemoptysis, headache, diarrhea, lymphocytopenia, and shortness of breath [5].

The renin-angiotensin aldosterone system (RAAS) consists of two principal mutually antagonistic axes: the angiotensin-converting enzyme/angiotensin II/angiotensin II type 1 receptor (ACE/AngII/AT1R) axis and the angiotensin-converting enzyme 2/angiotensin-(1-7)/mas receptor (ACE2/Ang(1-7)/MasR) axis [6]. ACE degrades Ang I to Ang II. The harmful effects of Ang II-mediated by AT1R are well recognized, such as pro-inflammation, fibrosis, elevated blood pressure, and cardiovascular damage [7]. ACE2 mainly degrades Ang II to the Ang-(1-7), which binds to Mas receptors and antagonizes the ACE/Ang II/AT1R axis [8]. Coronaviruses have many stinging glycoproteins on their surface. ACE2 is widely present as a receptor for proteins in the lung, kidney, testis, adipose, brain tissue, and vascular smooth muscle cells. The harmful effects of Ang II-mediated by AT1R are well recognized [9,10]. Viral entry into the human body acts on ACE2, leading to downregulation of ACE2 and substantial accumulation of Ang II, resulting in a range of deleterious effects as described above. Therefore, ACE2 is considered an important target for the virulence and entry efficiency of the SARS-CoV-2.

In addition to general supportive therapy and mechanical treatment of the respiratory system, a number of drugs are widely used in clinical management, such as angiotensin-converting enzyme inhibitors (ACEIs) and angiotensin II receptor blockers (ARBs). ACEIs reduce Ang II production by inhibiting ACE, and ARBs block the AT1R [11]. These drugs counteract the cytokine storm of SARS-CoV-2 and lung injury, and are also useful in treating comorbidities in patients with COVID-19 [12,13]. However, the use of these medications elevates transmembrane ACE2, thus increasing ACE2 expression in the presence of SARS-CoV-2. This leads to an increase in viral entry and replication in the body [14,15,16,17]. A controversial question was proposed, asking whether we should increase ACE2 levels in tissues, focus on suppressing the inflammatory response and treating comorbidities, or promote a decrease in ACE2 levels in tissues to reduce viral entry and replication.

The essence of addressing the above question is to examine the use of ACEIs and ARBs that is beneficial or detrimental to the outcomes ultimately exhibited by patients with COVID-19. Therefore, this article discusses the effect of ACEIs or ARBs use on mortality, severity, and healthcare resource utilization in patients with COVID-19 by including many previous studies and using meta-analysis methods from the perspective of evidence-based medicine (EBM). To enhance the objectivity and credibility of the data for the latter two outcome indicators, we quantified “severity” as “the number of intensive care unit (ICU) admissions” and “healthcare resource utilization” as “the length of hospital stays”.

## 2. Materials and Methods

### 2.1. Search Strategy

In this study, the English search terms “SARS-CoV-2, ACEIs, ARBs” and the related Chinese search terms were used to search the databases of CNKI, PubMed, Science, Cell, Springer, Nature, Web of Science, and Embase. The search period was from the establishment of each database to October 2022. Some new articles were added by tracing the cited literature of the retrieved articles.

### 2.2. Inclusions and Exclusions Criteria

Inclusions: (1) domestic and foreign published randomized controlled trials, cohort studies, retrospective studies, and case studies; (2) all patients tested positive for SARS-CoV-2 infection; (3) patients in the control group discontinued, did not take ACEI/ARB drugs or took other anti-hypertensive drugs. The experimental group took or continued to take ACEI or ARB drugs; (4) outcome indicators included at least one of the following: ① number of deaths; ② ICU admissions; ③ length of hospital stays. Exclusions: ① articles without randomized controlled trials; ② animal experiments; ③ duplicate publications; ④ articles with no access to original data; ⑤ review and hypothesis articles; ⑥ articles with randomized controlled trial content that did not match the study content; ⑦ articles with incomplete or severely missing data; ⑧ articles without any of the above-mentioned outcome indicators.

### 2.3. Literature Quality Assessment

The risk of bias in the included literature was evaluated using the Cochrane Collaboration (https://training.Cochrane.org/handbook (accessed on 24 November 2024)) for the included literature, judged by ① whether correct use of randomization method; ② whether allocation concealment was used properly; ③ whether blinding was used correctly for patients; ④ whether the study personnel proper use of blinding; ⑤ whether the outcomes, as well as data, were complete; ⑥ whether the outcomes were selectively reported; ⑦ whether there was relevant bias.

### 2.4. Data Extraction

The following characteristics of the control and experimental groups from the included literature were extracted for this study: first author’s name, number of people in the group, median or mean of age in the group, number of deaths in the group, length of hospital stays, and ICU admissions. It is worth mentioning that the software used in this study uses the mean and standard deviation (rather than the median) for the statistical treatment of continuous variables, so we used the method recommended by Luo [18] and Wan’s [19] method to estimate the mean and standard deviation using the median, the first quartile, the third quartile, and the sample size (the quantification of the continuous variables in some of the studies used the median rather than the mean).

### 2.5. Statistical Analysis

We performed a meta-analysis of mortality, ICU admissions, and length of hospital stays in the two groups using Review Manager 5.3 software. It was assumed that the study outcomes reporting the two groups of patients were independent. Due to the expected heterogeneity, data were analyzed using the random effects model. The model showed differences between studies. Meta-analysis was performed using the Mantel–Haenszel odds ratio for dichotomous data (mortality and ICU admissions). For continuous variables (length of hospital stays). Meta-analysis was performed using inverse variance standardized mean difference (SMD). All results were judged by values for correlation (p) and heterogeneity (I^2^); if I^2^ ≥ 50%, it is high heterogeneity, and if I^2^ < 50%, it indicates low heterogeneity.

### 2.6. Publication Bias of Assessment

Because the three indicators analyzed were included in more than ten articles, we used funnel plots to show the included essays’ publication bias visually. To make the results more objective, we used Egger’s regression to assess the bias results with *p*-value (*p* < 0.05) and intercept (the closer to 0, the smaller the risk of bias).

### 2.7. Sensitivity Analysis

Sensitivity analysis was performed on the literature included in the three indicators to test the reliability of the meta-analysis, using CMA 3.0 software with the “leave-one-out” to test whether each piece of literature had a significant effect on the combined effect. The results were quantitatively assessed using *p*-values. indicating that the likelihood of publication bias is almost non-existent.

### 2.8. Statement

The study was registered in the PROSPERO (CRD42023389240).

## 3. Results

### 3.1. Articles Search Results

**Figure 1:**
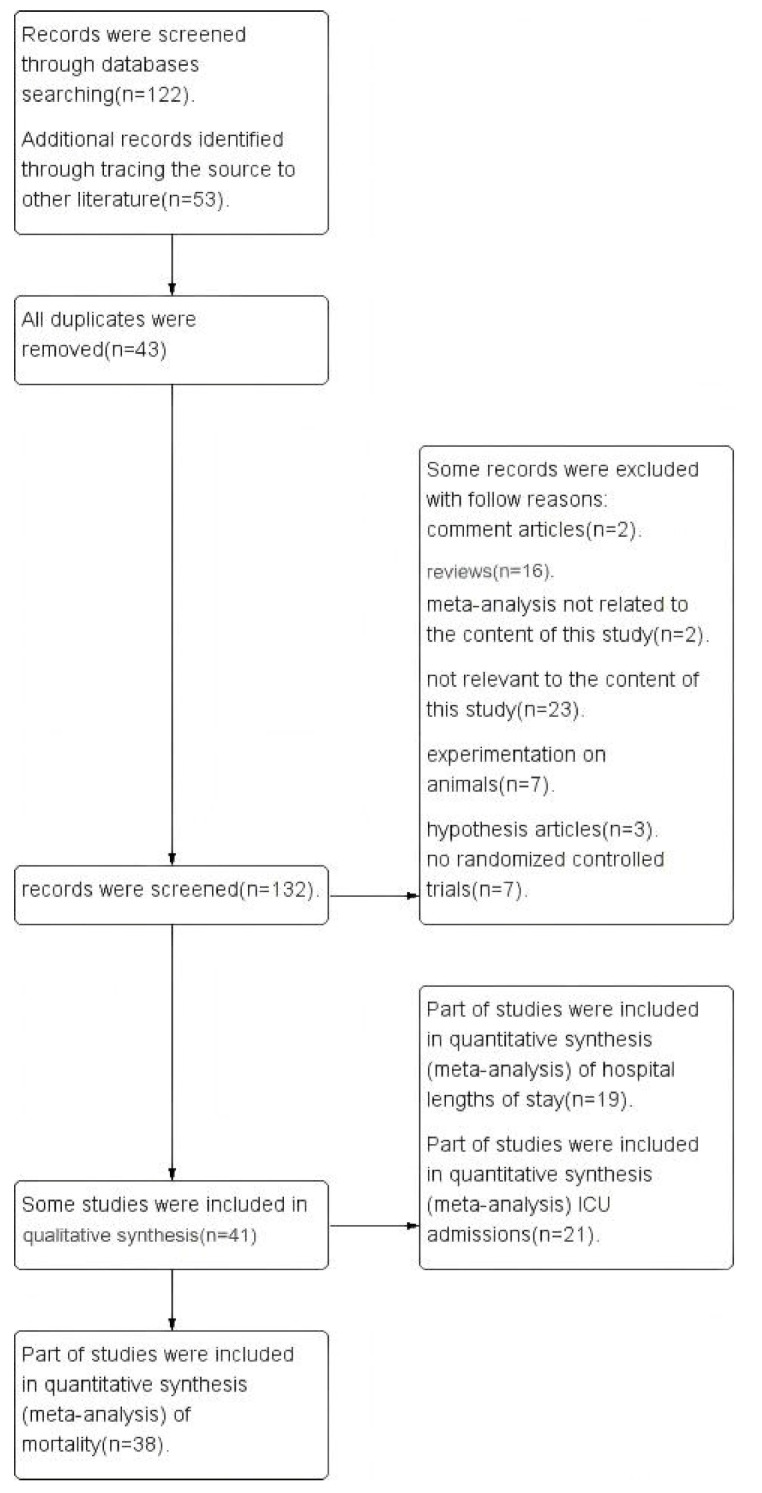
Selection process of meta-analyses (PRISMA) flow diagram.

A total of 122 papers were researched according to the above search strategy. In total, 53 papers were added by tracing the references in some studies, and 43 duplicates were deleted by using EndNote X9 software. In addition, 61 essays were excluded by reading the abstracts and introduction, and another 31 papers were excluded by reading the full text. A total of 40 papers were finally included, of which 38 were used for mortality analysis [20,21,22,23,24,25,26,27,28,29,30,31,32,33,34,35,36,37,38,39,40,41,42,43,44,45,46,47,48,49,50,51,52,53,54,55,56,57], 21 for analysis of the ICU admissions [21,22,23,24,26,27,29,30,33,35,36,37,38,39,40,41,44,45,54,58], and 19 for the analysis of the length of hospital stays [20,21,23,24,26,38,40,41,43,47,48,51,53,54,55,57,58,59,60] (Figure 1).

### 3.2. Basic Characteristics of the Included Studies

The included literature was in English, with 21,458 patients in the control group and 7300 patients in the experimental group for mortality, 587 patients in the control group and 484 patients in the experimental group for ICU admissions, and 2509 patients in the control group and 1681 patients in the experimental group for the length of hospital stays. Furthermore, the countries, the proportion of female patients in the sample, and some underlying diseases (hypertension, diabetes, cerebrovascular, and respiratory diseases) were extracted and analyzed (Table 1).

### 3.3. Quality Evaluation of the Included Literature

**Figure 2:**
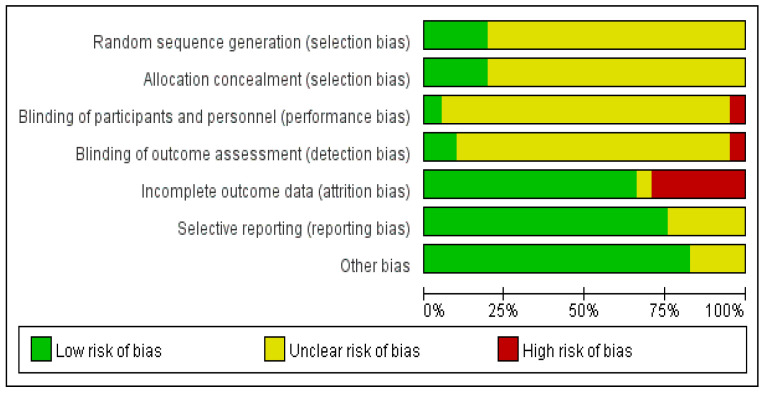
Bias risk assessed by the Cochrane assessment tool of included studies.

Among the included studies, eight articles described the method of random assignment sequence generation and concealment of the random assignment scheme, and two studies described the method of blinding subjects and experimenters, providing all information relevant to the validity of the blinding. Four papers blinded outcome assessors; data in thirty-nine articles were lost. Thirty-one articles had no selective reporting, and thirty-six papers had no other factors causing the risk of bias (Figure 2).

### 3.4. Meta-Analysis Outcomes

**Figure 3:**
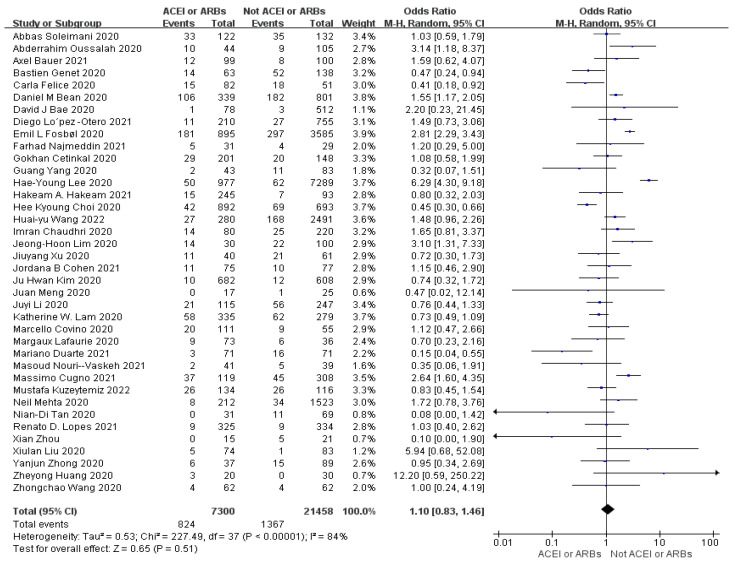
Forest plot for mortality (OR = 1.10, 95% CI 0.83–1.46, *p* = 0.43). The heterogeneity between studies was very high (I^2^ = 84%) [20,21,22,23,24,25,26,27,28,29,30,31,32,33,34,35,36,37,38,39,40,41,42,43,44,45,46,47,48,49,50,51,52,53,54,55,56,57].

**Figure 4:**
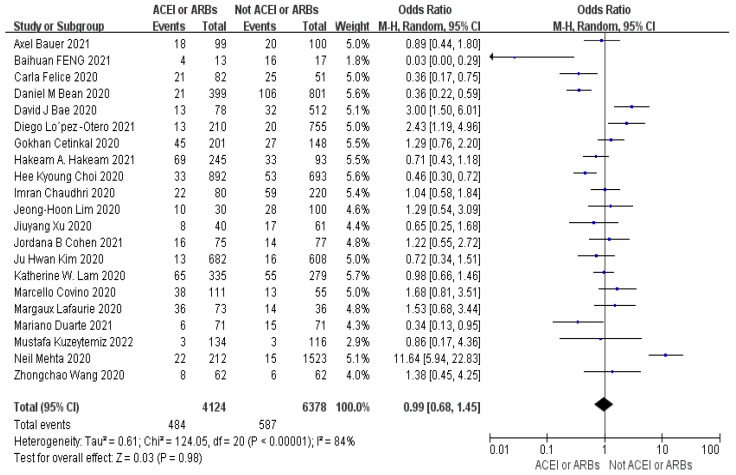
Forest plot for ICU admissions (OR = 0.99, 95% CI 0.68–1.45, *p* = 0.98). Heterogeneity was very high (I^2^ = 84%) [21,22,23,24,26,27,29,30,33,35,36,37,38,39,40,41,44,45,54,58].

**Figure 5:**
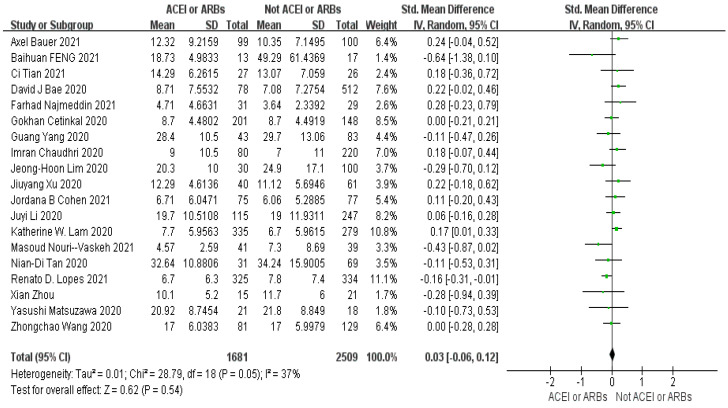
Forest plot for length of hospital stays (SMD = 0.03, 95% CI −0.06–0.12, *p* = 0.54). Heterogeneity was low (I^2^ = 37%) [20,21,23,24,26,38,40,41,43,47,48,51,53,54,55,57,58,59,60].

Meta-analysis indicates the heterogeneity ((I^2^ = 84%)) of mortality (OR = 1.10, 95% CI 0.83–1.46, *p* = 0.43) between studies was very high (Figure 3). For ICU admissions (OR = 0.99, 95% CI 0.68–1.45, *p* = 0.98). Heterogeneity was very high (I^2^ = 84%) (Figure 4). For the length of hospital stays (SMD = 0.03, 95% CI −0.06–0.12, *p* = 0.54). Heterogeneity was low (I^2^ = 37%) (Figure 5). In conclusion, the results of the meta-analysis showed that the use of ACEIs/ARBs was not associated with three outcome indicators of COVID-19 infections compared to not using ACEIs/ARBs. However, there is increasing heterogeneity in mortality and length of hospital stays between studies.

### 3.5. Meta-Regression Outcomes

**Figure 6:**
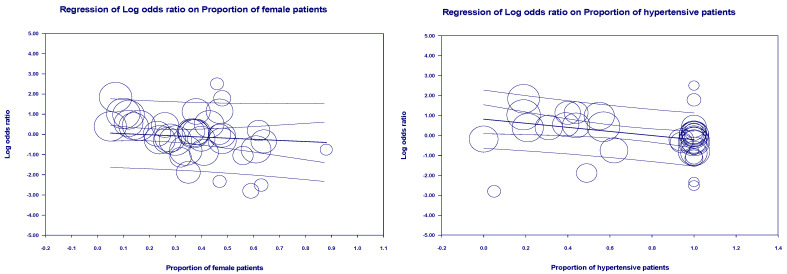

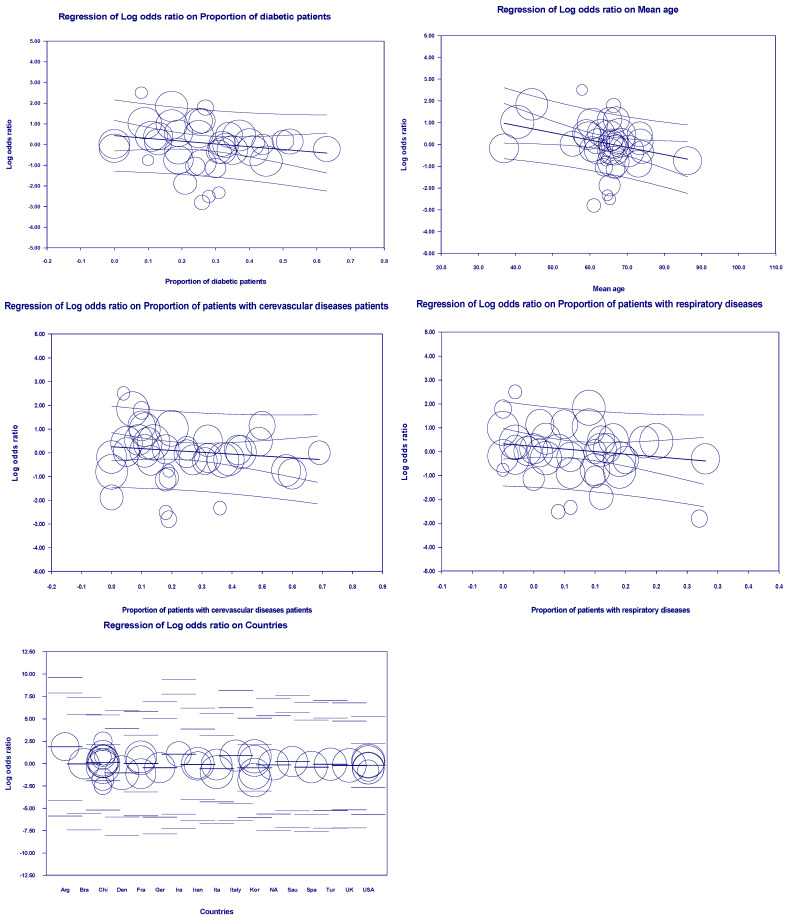
Univariate regression analysis for mortality.

Multivariate meta-regression was performed to explain variations in the association between mortality and being on ACEIs/ARBs revealed. Age means, the proportion of female patients in the sample, and the proportion of some underlying diseases (hypertension, diabetes, cerebrovascular, and respiratory diseases) in included studies covariates to be significant together and explained R^2^ = 63% of the between-study heterogeneity in mortality (*p* = 0.0000). The above multivariate was significant as a source of heterogeneity. We performed further univariate regression with mean age (*p* = 0.0124), the proportion of female subjects (*p* = 0.0013), the proportion of hypertensive patients (*p* = 0.0083), the proportion of diabetic patients (*p* = 0.1734), the proportion of cardiovascular patients (*p* = 0.3317) and the ratio of patients with respiratory system diseases (*p* = 0.2143), and the countries (*p* = 0.9630). Therefore, the proportion of female and hypertensive patients, and mean age, was the primary source of heterogeneity as seen by further univariate regression analysis (Figure 6).

**Figure 7:**
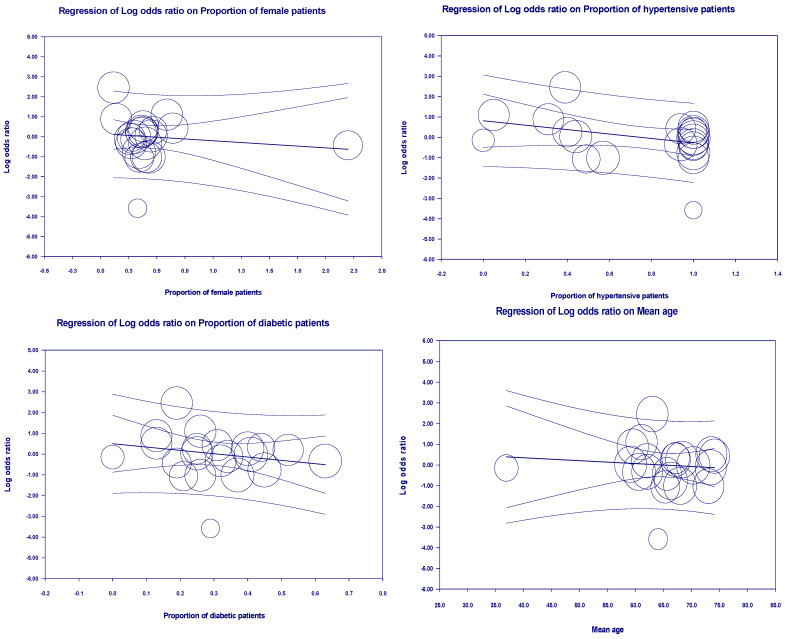

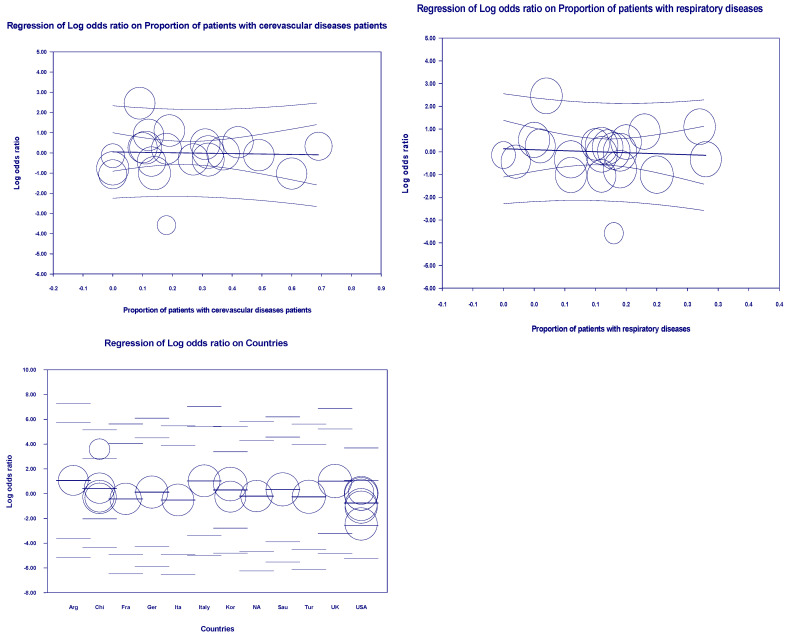
Univariate regression analysis for ICU admissions.

For ICU admissions, covariates are significant in included studies and explained R^2^ = 31% of the between-study heterogeneity (*p* = 0.0770). The above multivariate was not significant as a source of heterogeneity. We performed further univariate regression for mean age (*p* = 0.6623), the proportion of female subjects (*p* = 0.5185), the proportion of hypertensive patients (*p* = 0.0856), the proportion of diabetic patients (*p* = 0.3017), the proportion of cardiovascular patients (*p* = 0.8634) and the ratio of patients with respiratory system diseases (*p* = 0.7387), and the countries (*p* = 0.5508). Therefore, we did not find a specific source of heterogeneity. However, it was within the acceptable range due to its low heterogeneity (Figure 7).

**Figure 8:**
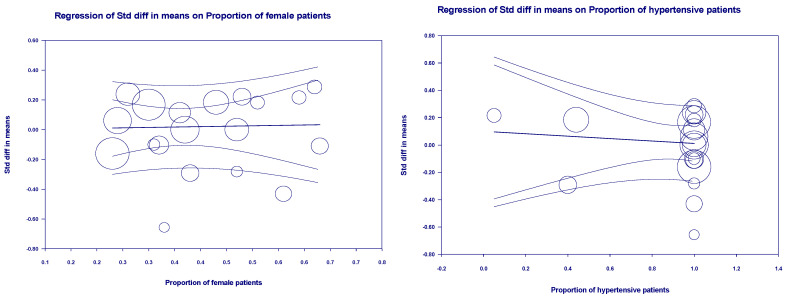

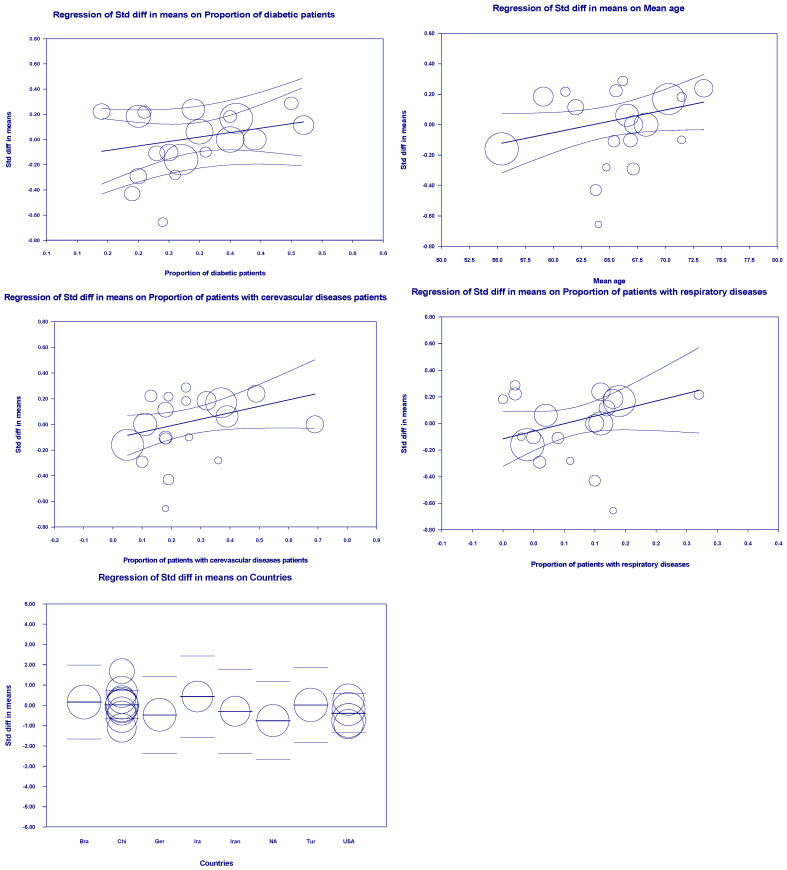
Univariate regression analysis for the length of hospital stays.

Moreover, covariates for the length of hospital stays are significant and only explained R^2^ = 100% of the between-studies heterogeneity (*p* = 0.3118 > 0.05). Mean age (*p* = 0.0368), the proportion of females (*p* = 0.8902), the proportion of patients with hypertension (*p* = 0.6701), the proportion of patients with diabetes (*p* = 0.2100), the proportion of patients with cardiovascular disease (*p* = 0.0312), and the ratio of patients with respiratory system diseases (*p* = 0.0597), and the countries (*p* = 0.6585). By further univariate regression we found that mean age and patients with cardiovascular disease are specific sources of heterogeneity (Figure 8).

### 3.6. Publication Bias

**Figure 9:**
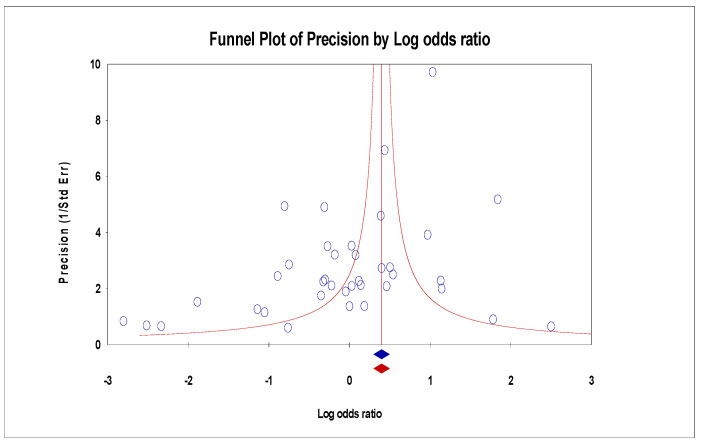
Funnel plots of mortality.

**Figure 10:**
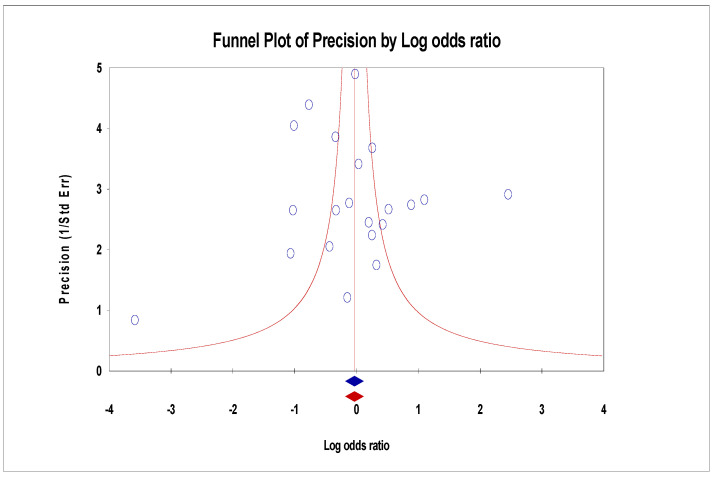
Funnel plots of ICU admissions.

**Figure 11:**
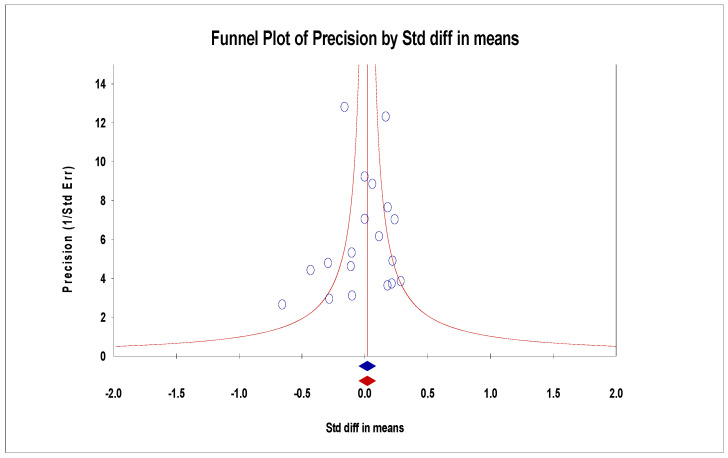
Funnel plots of length of hospital stays.

For the funnel plot, although the effect values for the number of deaths in each study were concentrated at the top of the graph and distributed on both sides of the total effect, initially indicating that the publication bias was not significant. However, Egger’s regression intercept was −1.92 (95% CI −3.60–0.60, *p* = 0.01), indicating some publication bias (Figure 9). For the ICU admissions, the funnel plot showed that the effect values were evenly distributed on both sides of the total effect, and the intercept of Egger’s regression was −0.44 (95% CI −1.83–0.95, *p* = 0.51). Both results indicated that there was no significant publication bias (Figure 10). The effect values for the literature included in the study of the length of hospital stays were also evenly distributed on both sides of the total effect and clustered at the top of the graph, with an intercept of 0.35 (95% CI −3.14–3.84, *p* = 0.84); also confirming that there was no significant publication bias in the included studies (Figure 11).

### 3.7. Sensitivity Results

**Figure 12:**
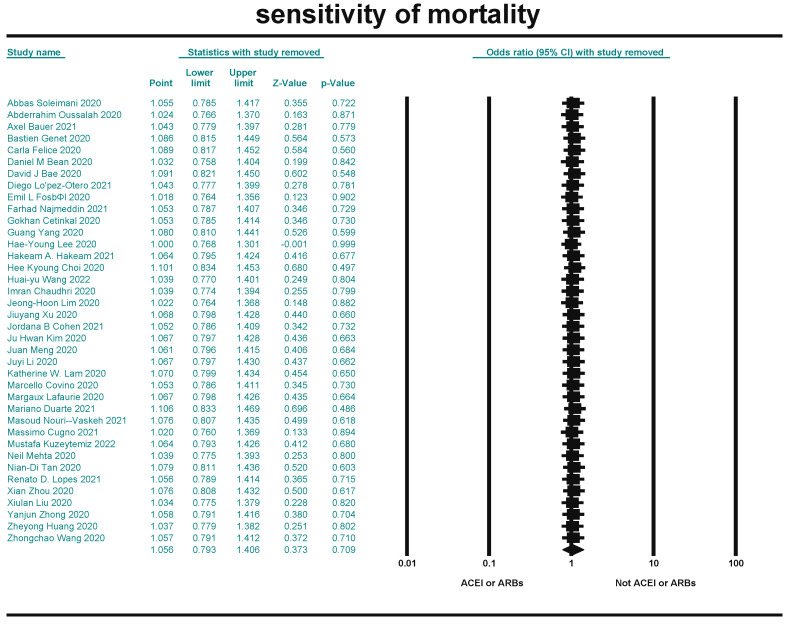
Sensitivity analysis for mortality [20,21,22,23,24,25,26,27,28,29,30,31,32,33,34,35,36,37,38,39,40,41,42,43,44,45,46,47,48,49,50,51,52,53,54,55,56,57].

**Figure 13:**
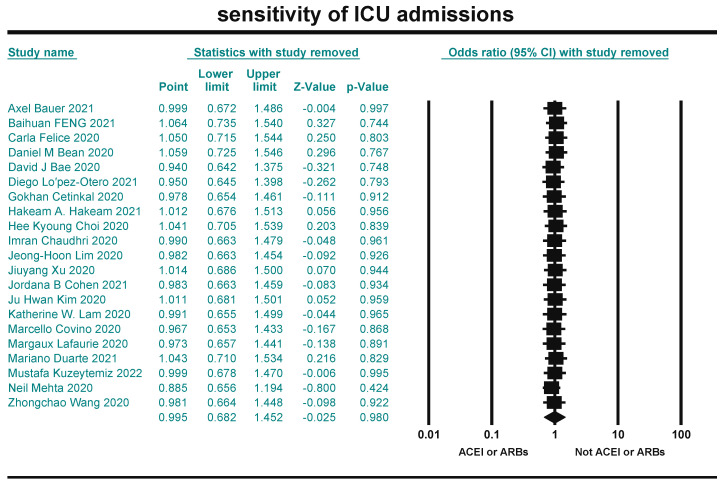
Sensitivity analysis for ICU admissions [21,22,23,24,26,27,29,30,33,35,36,37,38,39,40,41,44,45,54,58].

**Figure 14:**
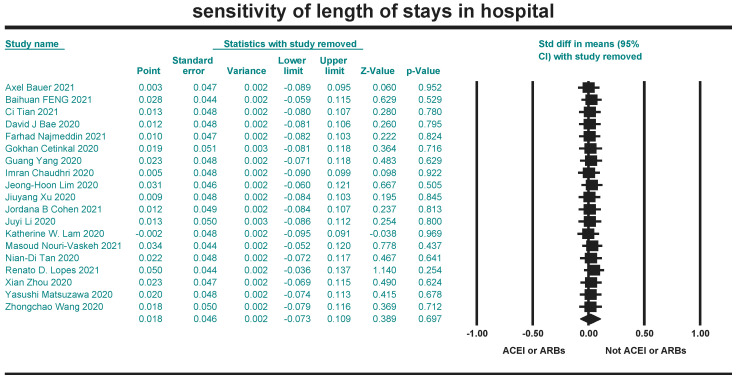
Sensitivity analysis for length of hospital stays [20,21,23,24,26,38,40,41,43,47,48,51,53,54,55,57,58,59,60].

Sensitivity analysis is a method to test the stability of the meta-analysis results. CMA 3.0 software was used to perform sensitivity analysis on the included literature of the three indicators by the leave-one-out method. The results were combined after excluding the included literature one by one. The effect on the outcome indicators was not significant, indicating that the results of this study were stable (Figure 12, Figure 13 and Figure 14).

## 4. Discussion

ACEIs and ARBs are widely utilized in the management of COVID-19 patients with multiple comorbidities, such as hypertension and ischemic heart disease [61]. On the one hand, ACEI inhibits ACE and reduces Ang I production, while ARBs act by blocking the action of Ang II on its pro-oxidant/pro-inflammatory receptor AT1. Thus, both types of drugs downregulate the activity of the RASS pro-inflammatory axis [62]. ACEIs and ARBs have also been shown to reduce lung injury in mouse models constructed in several previous studies [63]. The two drugs also significantly modified pulmonary fibrosis while treating different patients with viral pneumonia [64,65]. The above results are undoubtedly beneficial for the treatment of patients with COVID-19. However, ACEI also has significant drawbacks, such as a reduced effect of Ang II on the anti-inflammatory receptor AT2, in particular, ACEI can also induce an increase in bradykinin, which can participate in blood pressure regulation and inflammation by increasing vascular permeability and vasodilatory effects. It has a robust pro-inflammatory effect [11]. The pro-inflammatory effects of bradykinin may counteract ACEI-induced downregulation of the Ang II/AT1 pro-inflammatory axis [66]. More importantly, using both drugs promotes the expression of ACE2, which increases viral replication and viral load in the body and leads to more severe disease in patients with COVID-19. Therefore, it is necessary to investigate whether ACEIs or ARBs reduce the body’s inflammatory response and counteracts the cytokine storm in patients with COVID-19 or whether they tend to increase the amount of virus entering the body, causing exacerbation of the disease in patients with COVID-19. Our meta-analysis illustrates that the use of the two drugs does not affect these three outcome indicators from the perspective of whether the use of the two drugs adds to the mortality, severity, and healthcare resource utilization of COVID-19.

The results of our meta-analysis showed that the use of ACEIs and ARBs had no significant effect on mortality, severity, or healthcare resource utilization for patients with COVID-19, and the multivariate meta-regression model for mortality showed that 63% of the heterogeneity between studies could be explained by mean age, the proportion of female, countries, proportion patients of hypertensive, diabetes, cardiovascular disease, and respiratory disease explained. The results of further univariate meta-regression revealed that the variable proportion of females was the primary source of heterogeneity. The exact cause needs further study. The results of the multivariate regression analysis of the ICU admissions and length of hospital stays showed that the above-mentioned moderating variables were able to explain 31% and 100% of the fraction. The heterogeneity itself was low for the former, for which further univariate regression did not find a source of heterogeneity within an acceptable range, and for the latter, which had a very high heterogeneity (I^2^ = 84%). However, our model did not find a specific source of heterogeneity.

This study has some advantages: it had a larger sample size, stricter inclusion and exclusion criteria of studies, more consistent data, and more credible results. We also cited univariate and multivariate meta-regression models and discussed the sources of heterogeneity between studies. This analysis also contains some limitations: ① the data in some studies were incomplete. ② Medical records, which may be less reliable, screened for use of the two drugs. ③ High heterogeneity in the included studies due to variations in clinical studies, and to deal with this difficulty, we used meta-regression to explore the sources of heterogeneity. We identified some of the sources of inter-study heterogeneity, however, the reasons for the impact of heterogeneity on the results are not explored in this paper. ④ Ethnic differences in the included study samples may have created high heterogeneity between studies, but because the ethnic classification of the samples is not mentioned in the literature, we could not discuss it.

## 5. Conclusions

Due to the lack of statistical significance of the findings of the meta-analysis, it concluded that the use of ACEIs or ARBs was not significantly correlating with mortality, severity, and healthcare resource utilization in patients with COVID-19, which provides a clinical basis for safe drug use. More extensive clinical trial studies are expected to provide further relevant proof.

## Figures and Tables

**Table 1:** Basic characteristics of the included studies.

First Author	Countries	Y^p^	Mean Age	P^f^	P^h^	P^d^	P^c^	P^r^	Control Group				Experimental Group			
									No.	C^m^	C^l^, Mean, d	C^i^	No.	E^m^	E^l^, Mean, d	E^i^
Abbas Soleimani	Iran	2020	66.39	0.37	1.00	0.00	0.43	0.09	132	35	NA	NA	122	33	NA	NA
Abderrahim Oussalah	France	2020	65.34	0.47	0.44	0.26	0.50	0.10	105	9	NA	NA	44	10	NA	NA
Axel Bauer	Austria and Germany	2021	73.44	0.26	1.00	0.34	0.49	0.16	100	8	10.35	20	99	12	12.32	18
Baihuan FENG	China	2021	64.03	0.33	1.00	0.29	0.18	0.18	17	NA	49.29	NA	13	NA	18.73	NA
Bastien Genet	France	2020	86.28	0.61	0.62	0.19	0.58	0.15	138	52	NA	NA	63	14	NA	NA
Carla Felice	Italy	2020	73.02	0.41	1.00	0.26	0.60	0.11	51	18	NA	25	82	15	NA	21
Ci Tian	China	2021	71.46	0.51	1.00	0.40	0.25	0.00	27	NA	13.07	15	27	NA	14.29	NA
Daniel M Bean	UK	2020	67.97	0.16	0.57	0.37	0.14	0.25	801	182	NA	106	399	106	NA	21
David J Bae	USA	2020	45.98	0.59	0.05	0.26	0.19	0.32	512	3	7.08	13	78	1	8.71	7.2
Diego Lo’pez-Otero	Spain	2021	59.50	0.14	0.31	0.13	0.12	0.23	755	27	NA	20	210	11	NA	13
Emil L Fosbøl	Denmark	2020	40.52	0.10	0.19	0.09	0.20	0.14	3585	297	NA	NA	895	181	NA	NA
Farhad Najmeddin	Iran	2021	66.21	0.62	1.00	0.50	0.25	0.02	29	4	3.64	NA	31	5	4.71	4
Gokhan Cetinkal	Turkey	2020	68.28	0.37	1.00	0.40	0.11	0.16	148	20	8.70	27	201	29	8.70	45
Guang Yang	China	2020	66.90	0.32	1.00	0.30	0.18	0.05	83	11	29.70	NA	43	2	28.40	NA
Hae-Young Lee	Republic of Korea	2020	44.40	0.07	0.19	0.17	0.07	0.14	7289	62	NA	NA	977	50	NA	NA
Hakeam A. Hakeam	Saudi Arabia	2021	60.61	0.40	0.94	0.63	0.32	0.11	93	7	NA	33	245	15	NA	69
Hee Kyoung Choi	Republic of Korea	2020	66.31	0.34	1.00	0.45	0.00	0.19	693	69	NA	53	892	42	NA	33
Huai-yu Wang	China	2022	65.00	0.05	0.21	0.11	0.05	0.02	2491	168	NA	NA	280	27	NA	NA
Imran Chaudhri	USA	2020	59.11	0.43	0.44	0.25	0.32	0.18	220	25	7.00	59	80	14	9.00	22
Jeong-Hoon Lim	Republic of Korea	2020	67.14	0.38	0.40	0.25	0.10	0.06	100	22	24.90	28	30	14	20.30	10
Jiuyang Xu	China	2020	65.60	0.48	1.00	0.19	0.13	0.02	61	21	11.12	17	40	11	12.29	8
Jordana B Cohen	7 countries	2021	62.00	0.36	1.00	0.52	0.18	0.17	77	10	6.06	14	75	11	6.71	16
Ju Hwan Kim	Republic of Korea	2020	62.09	0.27	1.00	0.32	0.27	0.33	608	12	NA	16	682	10	NA	13
Juan Meng	China	2020	64.30	0.88	1.00	0.10	0.19	0.00	25	1	NA	NA	17	0	NA	NA
Juyi Li	China	2020	66.60	0.24	1.00	0.35	0.39	0.07	247	56	19.00	56	115	21	19.70	NA
Katherine W. Lam	USA	2020	70.30	0.30	1.00	0.41	0.37	0.19	279	62	6.70	55	335	58	7.70	65
Marcello Covino	Italy	2020	73.55	0.38	1.00	0.13	0.42	0.05	55	9	NA	13	111	20	NA	38
Margaux Lafaurie	France	2020	74.09	0.64	0.94	0.31	0.31	0.20	36	6	NA	14	73	9	NA	36
Mariano Duarte	Argentina	2021	65.30	0.35	0.49	0.21	0.00	0.16	71	16	15.00	15	71	3	9.00	6
Masoud Nouri-Vaskeh	Iran	2021	63.79	0.56	1.00	0.24	0.19	0.15	39	5	7.30	NA	41	2	4.57	NA
Massimo Cugno	Italy	2021	60.70	0.12	0.55	0.17	0.11	0.00	308	45	NA	NA	119	37	NA	NA
Mustafa Kuzeytemiz	USA	2022	36.92	0.28	0.00	0.00	0.00	0.00	116	26	NA	3	134	26	NA	3
Neil Mehta	USA	2020	63.00	0.12	0.39	0.19	0.09	0.07	1523	34	NA	15	212	8	NA	22
Nian-Di Tan	China	2020	65.42	0.63	1.00	0.28	0.18	0.09	69	11	34.24	NA	31	0	32.64	NA
Renato D. Lopes	Brazil	2021	55.39	0.23	1.00	0.32	0.05	0.04	334	9	7.80	NA	325	9	6.70	NA
Xian Zhou	China	2020	64.74	0.47	1.00	0.31	0.36	0.11	21	5	11.70	NA	15	2	10.10	NA
Xiulan Liu	China	2020	66.39	0.48	1.00	0.27	0.10	0.00	83	1	NA	NA	74	5	NA	NA
Yanjun Zhong	China	2020	66.31	0.48	1.00	0.33	0.25	0.06	89	15	NA	NA	37	6	NA	NA
Yasushi Matsuzawa	Japan	2020	60	0.40	1.00	0.167	0.06	0.00	18	NA	21.80	NA	21	NA	20.92	NA
Zheyong Huang	China	2020	57.90	0.46	1.00	0.08	0.04	0.02	30	0	NA	NA	20	3	NA	NA
Zhongchao Wang	China	2020	67.18	0.47	1.00	0.44	0.69	0.15	62	NA	17.00	6	62	4	17.00	8

Abbreviations: Y^p^, year of publication; P^f^, proportion of females; P^h^, proportion of patients with hypertension; P^d^, proportion of patients with diabetes; P^c^, proportion of patients with cardiovascular diseases; P^r^, proportion of patients with respiratory system diseases; No, number; C^m^, control group mortality; C^l^, length of hospital stays in control group; C^i^, number of ICU admissions in control group; E^m^, experimental group mortality; E^l^, length of hospital stays in experimental group; E^i^, number of ICU admissions in experimental group. NA indicates that this value was not recorded and this article will not be used for a meta-analysis of this outcome indicator. The software used in this study uses the mean and standard deviation (rather than the median) for the statistical treatment of continuous variables, so we used the method recommended by Luo [18] and Wan’s [19] method to estimate the mean and standard deviation using the median [20,21,22,23,24,25,26,27,28,29,30,31,32,33,34,35,36,37,38,39,40,41,42,43,44,45,46,47,48,49,50,51,52,53,54,55,56,57,58,59,60].

## Data Availability

Not applicable.
